# Radiation Response of Negative Gate Biased SiC MOSFETs

**DOI:** 10.3390/ma12172741

**Published:** 2019-08-27

**Authors:** Akinori Takeyama, Takahiro Makino, Shuichi Okubo, Yuki Tanaka, Toru Yoshie, Yasuto Hijikata, Takeshi Ohshima

**Affiliations:** 1 National Institutes for Quantum and Radiological Science and Technology, 1233 Watanuki-machi, Takasaki 370-1292, Japan; 2 Sanken Electric Co., Ltd., Niiza, Saitama 352-8666, Japan; 3 Graduate School of Science and Engineering, Saitama University, Saitama 338-8570, Japan

**Keywords:** SiC, MOSFET, gamma-rays, radiation response, gate bias

## Abstract

Silicon carbide (SiC) metal-oxide-semiconductor field effect transistors (MOSFETs) are expected as power electronic devices for high radiative conditions, including nuclear plants and space. Radiation response of commercial-grade prototype SiC MOSFETs with applying the gate bias is of interest, in terms of installation of the device in robots or sensors working under such radioactive circumstances. Due to gamma-rays irradiation, the threshold voltages (*V*_th_) of samples with un- and negative-biased up to −4.5 V slightly shift toward the negative voltage side. In contrast, the positive bias of 2.25 V shifts *V*_th_ more negatively. Positive charge densities trapped in the gate oxide of un- and positive-biased samples increased with increasing dose. However, no significant increase was observed for negative-biased samples of −2.25 and −4.5 V. We calculated characteristic parameters for the accumulation of holes in the gate oxide, σ_p_*J*_p_ which is defined as the product of current density due to holes generated by irradiation and capture cross section for a hole in a trap, and it is lower for these negative biased samples compared with the unbiased case. Application of appropriate negative gate biases to SiC MOSFETs during irradiation suppresses accumulation of positive charges in the gate oxide and negative shift of *V*_th_, due to irradiation.

## 1. Introduction

Owing to its wide band gap and strong covalent bond of silicon carbide (SiC), SiC metal-oxide-semiconductor field effect transistors (MOSFETs) are known as power electronic devices with a higher radiation tolerance. The radiation response of SiC MOSFETs is of interest in terms of the application of this device for use in radiative conditions, including nuclear plants and space. In particular, for the decommissioning of Tokyo Electric Power Company Holdings (TEPCO) Fukushima Dai-ichi nuclear plants, although operations of robots or sensors under high radioactive circumstance are required [1,2,3], commercial Si devices seem to be susceptible to radiation damage in such harsh conditions [4,5]. Recently, the radiation response of commercially available or prototype SiC MOSFETs against gamma ray irradiation was studied by several authors [6,7,8,9,10,11,12]. These MOSFETs have a channel length shorter than 1 μm and width on the order of a meter to conduct large currents. Also, specific processes are employed to improve electric properties, including nitridation of the interface between the oxide and SiC. These characteristics of commercial or prototype SiC MOSFETs might make their electrical properties be more sensitive to irradiation compared with ones for the basic research. So far, the radiation response was studied in terms of device structure and fabrication process [6], irradiation conditions, such as high temperature and humidity [7,8,10], and application of gate bias [11,12]. In particular, applying the positive gate bias during irradiation, the threshold voltage (*V*_th_) remarkably shifts in the negative direction, as reported for irradiation of positively biased Si MOSFETs [13,14,15]. It is explained that the radiation-induced holes move toward the Si/oxide (SiO_2_) interface due to the applied electric field and are trapped by defects in the oxide near the interface [16,17,18]. In addition, varying the gate bias during irradiation from positive to either negative or zero volts results in substantial recovery of *V*_th_ [11]. These findings indicate that annihilation of holes trapped in the gate oxide takes place by changing the polarity of the bias [19,20,21,22]. Such radiation induced charge neutralization for Si MOS devices was also explained in terms of direct annealing of trapped holes and charge compensation of positive charges of trapped holes by electrons [23]. Contribution of the charge compensation to the annealing of net positive charges is less significant for non-hardened oxide, since it involves a larger amount of oxygen vacancies as a hole trap, resulting from dry or high temperature oxidation process [16]. In contrast to positive bias, the effect of negative gate bias on the radiation response of commercial or prototype SiC MOSFETs is still unclear. Although it has been suggested that negative gate bias results in the annihilation of accumulated holes [11], more detailed research is required for practical use and to determine the condition in which annihilation takes place. In addition, a study about the radiation response under a negative biased condition is also of interest to predict aged electrical properties of SiC MOSFETs in a non-radiative condition. Since gamma-rays can generate a number of additional holes/electrons in the oxide/semiconductor, irradiation serves as an accelerated test and allows us to avoid time-consuming experiments [24]. Previous reports about Si MOSFETs showed that long term degradation behavior under bias and temperature stress is quite similar to that caused by irradiation. In this report, the effect of negative gate bias on the radiation response of prototype SiC MOSFETs is studied. Dependence of annihilation and accumulation behaviors of holes on the applied bias is revealed and the condition in which annihilation predominantly takes place is determined.

## 2. Materials and Methods

The samples used in this study were vertical 4H-SiC MOSFETs mounted in “TO3P” packages, provided by Sanken Electric Co., Ltd. (Niiza, Japan). The blocking voltage and the rated current are 1200 V and 20 A, respectively. These were prototype devices, although fabricated using commercial processes, such as nitridation of the gate oxide. Dry oxidation followed by 10% N_2_O treatment was carried out to fabricate a gate oxide of 45 nm thickness. P-wells in the channel region were formed by ion implantation. Doping concentration of acceptor was approximately 1 × 10^16^−2 × 10^16^ cm^−3^. The samples were irradiated with gamma-rays from ^60^Co at a dose rate of 10 kGy (SiO_2_)/h up to 2 MGy at room temperature (RT) in dry N_2_ atmosphere. During irradiation, negative biases of −9, −4.5, and −2.25 V were applied to the gate electrode, and source and drain electrodes were grounded. For comparison, SiC MOSFETs were also irradiated with the gate node grounded and with a positive bias of +2.25 V. At the intervals between each irradiation, drain current (*I*_D_)–gate voltage (*V*_G_), and *I*_D_–drain voltage (*V*_D_), characteristics were measured at RT using a semiconductor parameter analyzer (Agilent 4156 B).

Densities of the positive charge trapped in gate oxide and negative charge at the interface of SiC/oxide were calculated on the slope of *I*_D_–*V*_G_ curves [25]. The midgap current *I*_mg_ was calculated using the equation representing ideal drain current in the subthreshold region [26]:

(1) $$I_{D}=2^{\frac{1}{2}}\;\mu \left(\frac{W}{2L}\right)\left(\frac{qN_{A}L_{B}}{\beta }\right){\left(\frac{n_{i}}{N_{A}}\right)}^{2}e^{{\beta \Psi }_{s}}{\left({\beta \Psi }_{s}\right)}^{-\frac{1}{2}}$$

where *W*, *L*: channel width and length, *q*, *N*_A_, *n*_i_, Ψ_s_: the elementary charge, acceptor concentration in channel region, intrinsic carrier concentration at the absolute temperature (*T*), and band bending at the surface of SiC, respectively. Channel size and acceptor concentration data was provided by Sanken Electric Co., Ltd. The reciprocal thermal energy (=*q*/*k*_B_*T*, where *k*_B_ is Boltzmann constant) is β and Debye length (=(ε_sic_·ε_0_/(β*qN*_A_))^1/2^, where ε_sic_, ε_0_ are the relative permittivity of SiC and permittivity in vacuum, respectively) is *L*_B_. Effective mobility (μ) is calculated from linear region of *I*_D_–*V*_D_ curves. When the surface is bending, Ψ_s_ is equal to Ψ_b_ = (*k*_B_*T*/*q*) ln(*N*_A_/*n*_i_) in the above equation, the midgap current *I*_mg_ was obtained. Physical parameters, including the dielectric constant ε_sic_ of 9.7, and the intrinsic carrier concentration *n*_i_ at *RT* of 5.44 × 10^–9^ cm^–3^ were adopted from the reference [27]. The subthreshold region of *I*_D_–*V*_G_ curves was extrapolated to the calculated *I*_mg_ to obtain the midgap voltage, *V*_mg_. The extrapolation of *I*_D_–*V*_G_ curves was carried out carefully to exclude the influence of leakage current. For a sample before irradiation, the curve could be extrapolated from *I*_D_ = 10^−10^ A, however, for irradiated samples, the curve was extrapolated from *I*_D_ = 10^−7^ A. Finally, threshold voltage shift (Δ*V*_th_) of irradiated samples was separated into two terms of charge trapped in oxide (Δ*V*_ot_) and at interface (Δ*V*_int_), depicted as follows:

Δ*V*_th_ = Δ*V*_ot_ + Δ*V*_int_,  Δ*V*_ot_ = Δ*V*_mg_,  Δ*V*_int_ = Δ*V*_th_ − Δ*V*_mg_ (2)

where Δ*V*_mg_ is the shift of *V*_mg_ due to irradiation.

The areal densities of trapped holes Δ*N*_ot_ and interface state Δ*N*_int_ are obtained from equations:

(3) $$\Delta N_{\mathrm{ot}}\;=\;\frac{C_{\mathrm{ox}}}{q}\Delta V_{\mathrm{ot}},\;\;\;\;\Delta N_{\mathrm{int}}\;=\;\frac{C_{\mathrm{ox}}}{q}\Delta V_{\mathrm{int}}$$

where *C*_ox_ is oxide capacitance. Before estimation, we measured the high frequency (1 MHz) capacitance (*C*)–voltage (*V*) curves of MOS capacitors provided by Sanken Electric at room temperature (data are not shown). The thickness and fabrication process of the oxide is the same as that of the gate oxide of the MOSFETs used in this study. Calculated dielectric constant from oxide capacitance allows us to consider the gate oxide as SiO_2_. Accumulation and annihilation behavior of holes in the oxide due to irradiation is described by the following equation [22,28]:

(4) $$\frac{\partial p_{t}}{\partial t}\;=\;\sigma_{p}J_{p}\left(N_{t}\;-\;p_{t}\right)\;-\;\sigma_{n}J_{n}p_{t}$$

where *p*_t_ is hole density trapped at the defect, *J*_p,n_ is current density due to holes and electrons, σ_p,n_ is capture cross section for a hole in a trap and electron in trapped holes, respectively. *N*_t_ is the hole trap density. When the oxide electric field *E*_ox_ is positive, holes diffuse in the gate oxide from the gate electrode toward SiC/oxide interface and electrons diffuse in the opposite direction. The effect of detrapping of captured holes into SiO_2_ valence band [29] is also included in the second term of Equation (4). As previously reported, the gate oxide of practical SiC MOSFETs includes nitrogen-related defects [30] in addition to the oxygen vacancy, which is a popular positive charge trap in Si MOSFETs [31]. Thus, we evaluate accumulation and annihilation behavior of holes using Equation (4) in dose region below 100 kGy, in which the effect of nitrogen-related defects is not significant [6]. The oxide electric field during irradiation is approximated by dividing the sum of applied bias and the built-in voltage applied to the oxide at *V*_G_ = 0 V, by the oxide thickness. That is calculated by the following procedure. Using experimentally obtained *I*_D_ at *V*_G_ = 0 V from *I*_D_–*V*_G_ curve and Equation (1), surface potential Ψ_s_ at *V*_G_ = 0 V is obtained. Assuming electrical properties of irradiated SiC MOSFETs are predominantly determined by oxide trapped (*V*_ot_) and interface trapped charges (*V*_int_), *V*_G_ is correlated with Ψ_s_ and flat band voltage (*V*_FB_) as follows:

(5) $$V_{G}\;=\;V_{\mathrm{FB}}\;+\;V_{\mathrm{ot}}\;+\;V_{\mathrm{int}}\;+\;\Psi_{s}\;+\;\sqrt{\left(2\varepsilon_{\mathrm{SiC}}\varepsilon_{0}qN_{A}\Psi_{s}\right)}/C_{\mathrm{ox}}$$

After subtracting obtained Ψ_s_ from both side of the equation at *V*_G_ = 0 V, the right-hand side of the equation corresponds to the voltage applied to the oxide before applying the bias.

## 3. Results

**Figure 1:**
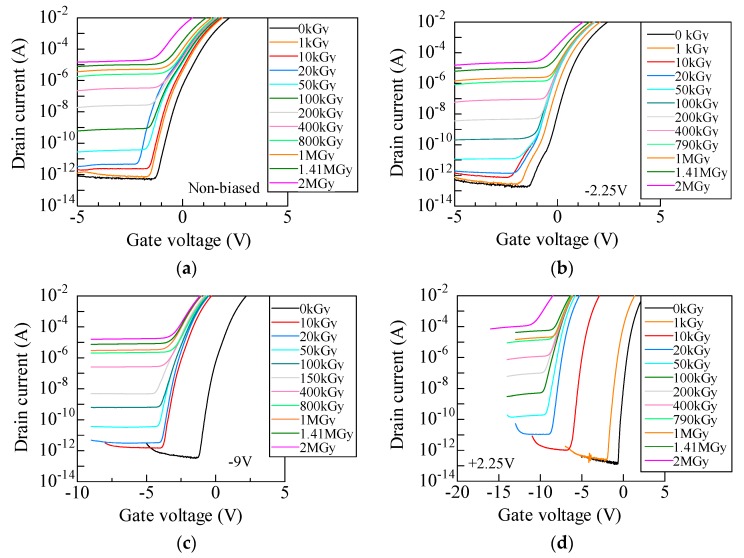
*I*_D_–*V*_G_ curves in the subthreshold region for irradiated SiC MOSFETs for (**a**) unbiased, (**b**) −2.25 V, (**c**) −9 V, and (**d**) +2.25 V. These were measured applying a drain voltage of 10 V.

Figure 1a–d show *I*_D_–*V*_G_ curves in the subthreshold region for unbiased, −2.25, –9, and +2.25 V biased samples. During the measurement, 10 V of *V*_D_ was applied. *I*_D_–*V*_G_ curves for negatively and unbiased samples (Figure 1a–c) negatively shift due to irradiation, however, no significant shift is observed above 20 kGy. It should be mentioned that an increase in leakage current with increasing dose is attributed to the leakage path generated by parasitic bias due to accumulated positive charges in the field oxide, rather than the degradation of the interface between SiC/oxide [10]. Since this study focuses on charge generated in the gate oxide, we do not discuss this leakage current in this study. In contrast, *I*_D_–*V*_G_ curves for +2.25 V shift more negatively at even 10 kGy irradiation.

**Figure 2:**
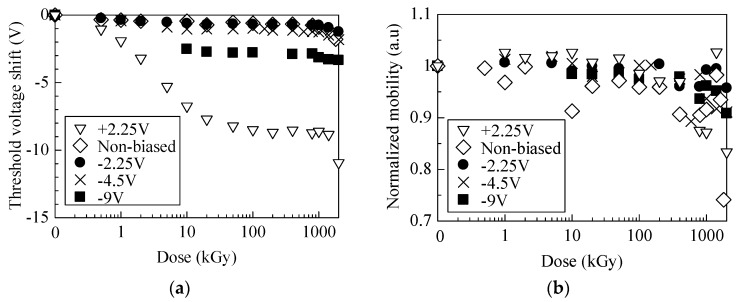
Plots of (**a**) the threshold voltage shift and (**b**) normalized mobility as a function of dose.

The threshold voltage shift Δ*V*_th_ is plotted as a function of dose. *V*_th_ is calculated by linear extrapolation of the square root of *I*_D_–*V*_G_ curves in the saturation region. Δ*V*_th_ is the voltage shift from the initial *V*_th_ of each sample before irradiation. In Figure 2a, Δ*V*_th_ of unbiased and negative biased samples slightly decreased up to 20 kGy and the +2.25 V biased sample decreased up to 50 kGy, and further negative shift is observed over 1 MGy. Observed shift above 1 MGy was caused by an increase of the density of hole traps in the oxide, probably as a result of cleavage of the Si–N bond [6].

In order to investigate the effect of interface traps on electrical properties, normalized channel mobilities are plotted as a function of dose in Figure 2b. Normalized channel mobility is defined as a ratio of the effective mobility for irradiated samples (μ_eff_), which was calculated from drain current (*I*_D_)–drain voltage (*V*_D_) curves, to compare with the initial (before-irradiated) sample (μ_eff0_). Each normalized mobility slightly decreased with the increasing dose. It resulted from electron scattering by increased shallow traps located at the interface between SiC/oxide [32]. However, at least below 200 kGy, significant degradation of mobility was not observed. It suggests that most of interface traps formed in this dose region were located far from the conduction band edge of SiC. Usually, charges trapped at such deep traps might also behave as scattering centers of carriers in the inversion layer. However, in the case of SiC MOSFETs, they are easily screened by the carriers, since the number of electrons in the inversion layer increases with increasing the gate voltage [33,34]. Although we cannot quantify contribution of the interface traps to the mobility degrade, deep traps act primarily as negative fixed charges in dose regions as low as several hundred kGy.

## 4. Discussion

**Figure 3:**
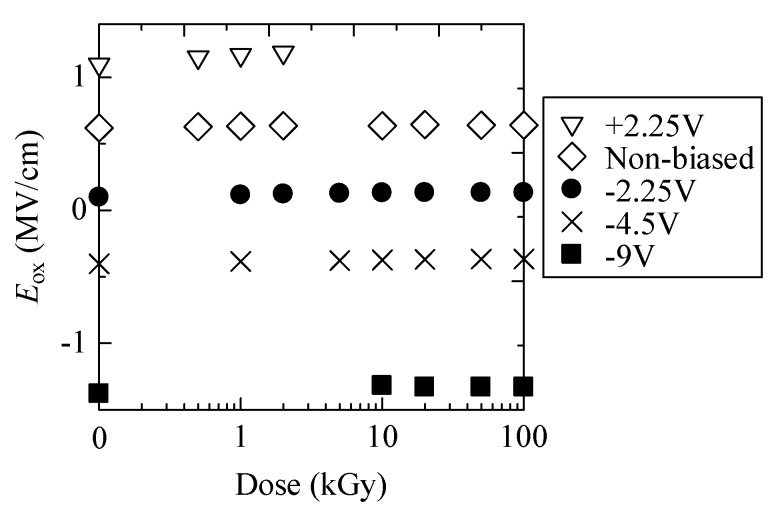
Plots of electric field gradient in the oxide during irradiation (*E*_ox_) as a function of dose.

Figure 3 is the estimated electric field formed in the gate oxide (*E*_ox_) during irradiation. For unbiased and negative biased cases, *E*_ox_ slightly increased up to 10 kGy and no significant change was observed with further irradiation. As shown in Figure 3, *E*_ox_ for −2.25 V is approximately 0 and *E*_ox_ for −4.5 V is slightly negative. For +2.25 V, we cannot obtain values above 5 kGy since large negative shift of *I*_D_–*V*_G_ curves inhibits to calculate Ψ_s_ at *V*_G_ = 0 V.

**Figure 4:**
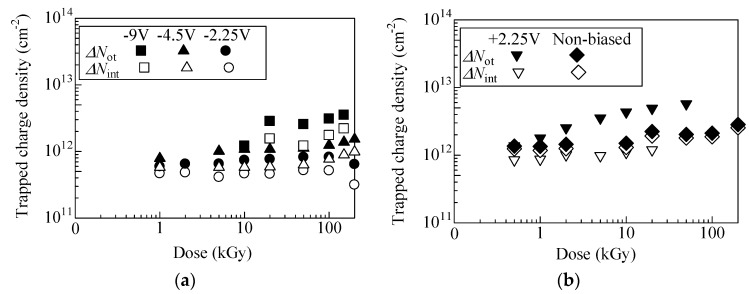
Positive charges trapped in the oxide (Δ*N*_ot_) and at the interface (Δ*N*_int_) for (**a**) negative biased, (**b**) un- and +2.25 V biased samples.

Figure 4a,b show charge densities trapped in oxide (Δ*N*_ot_) and at interface (Δ*N*_int_) plotted as a function of dose. Both Δ*N*_ot_ and Δ*N*_int_ are plotted up to 200 kGy, since large leakage current inhibits extrapolation of the *I*_D_–*V*_G_ curves. In the figures, an increase of Δ*N*_ot_ with an increasing dose resulted from accumulation of holes in the oxide. Δ*N*_ot_ for negative and unbiased samples gradually increased, while Δ*N*_ot_ of +2.25 V monotonically increased. Relative to Δ*N*_ot_, a smaller increase of Δ*N*_int_ with increasing dose was observed for every sample. The effect of application of the gate bias during irradiation on the interface property is smaller than that for the oxide.

**Figure 5:**
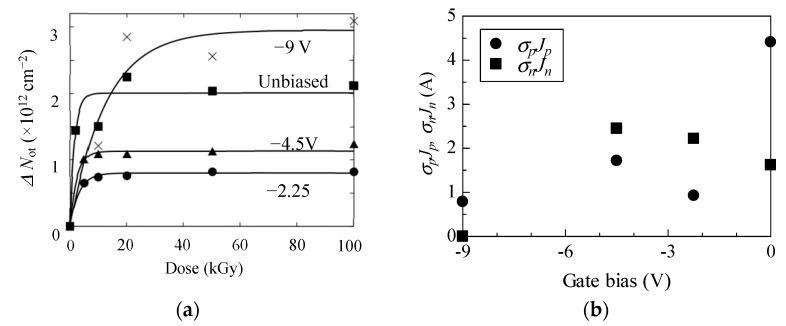
(**a**) Experimental plots and simulated curves for −2.5, −4.5 V, and non-biased samples plotted as a function of dose. (**b**) Estimated σ_p_*J*_p_ and σ_n_*J*_n_ plotted against applied gate bias during irradiation.

Accumulation and annihilation behavior of holes in the oxide were investigated by least square fitting of obtained Δ*N*_ot_ curves. On the basis of Δ*N*_ot_ of +2.25 V biased sample below 2 kGy, the density of hole trap in the oxide, *N*_t_, was calculated using Equation (4). In such a low dose region, the annihilation effect (second term of Equation (4)) could be negligible. Least square fitting of plots of Δ*N*_ot_ gives *N*_t_ of 2.7 × 10^12^ cm^2^, which is somewhat smaller than the reported *N*_t_ of 4.50 and 8.50 × 10^12^ cm [22]. Figure 5a shows experimental plots and estimated curves of Δ*N*_ot_ for unbiased and negative biased samples. We maintain the view that Δ*N*_ot_ estimated from experimental results essentially follow Equation (4), although the experimental uncertainties should be improved. Estimated σ_p_*J*_p_ and σ_n_*J*_n_ are also plotted as a function of the gate bias in Figure 5b, respectively. σ_n_*J*_n_ of negative biased samples slightly increased for −2.25 and −4.5 V biased cases relative to unbiased case. Since σ_n_*J*_n_ is annihilation probability of a trapped hole, this indicates that annihilation behavior was not significantly affected by application of these negative biases, while σ_p_*J*_p_ is less than half of the unbiased one. As the absolute value of the oxide electric field (*E*_ox_) for these negative biased samples was smaller than the unbiased case, as shown in Figure 3, the yield of hole due to irradiation is expected to be smaller [20]. Additionally, smaller *E*_ox_ might suppress movement of generated holes toward SiC/oxide interface, at which many hole traps exist. Thus, for −2.25 and −4.5 V biased cases, relatively lower σ_p_*J*_p_ was obtained. Further increasing the negative bias up to −9 V, *E*_ox_ becomes larger, as seen in Figure 3. Nevertheless, remarkable increase of σ_p_*J*_p_ was not observed and the annealing effect was not extracted from the fitting results. These suggest that most of the hole traps are located close to the SiC/SiO_2_ interface [35]. Due to the suppression of movement of holes toward the interface by negatively large *E*_ox_, a portion of holes generated by irradiation could be trapped. Low annealing effect is also interpreted as formation of traps too far from the gate electrode. They were beyond the reach of electrons coming from the gate electrode.

**Figure 6:**
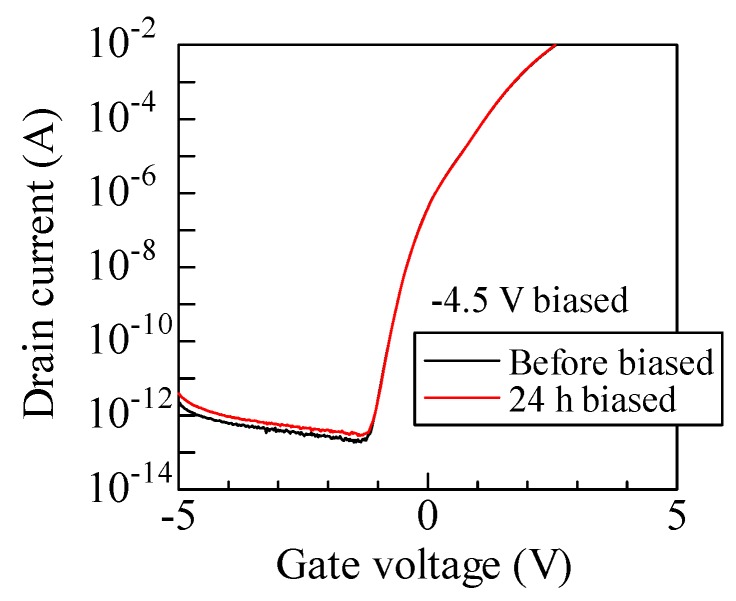
*I*_D_–*V*_G_ curves in the subthreshold region for biased SiC MOSFETs without irradiation. For *I*_D_–*V*_G_ measurement, drain voltage of 10 V were applied.

In this study, electrical properties of irradiated MOSFETs were characterized after each irradiation. It takes inevitably several minutes before the measurement is carried out. Such a relatively long interval is not recommended in terms of precise evaluation of the instability of *V*_th_ [36]. Due to the long interval, the amount of charges with a fast detrapping time constant cannot be evaluated by our measurement procedure [35]. In other words, obtained results in this study show the effect of charges with a slow time constant on the radiation response of MOSFETs. Furthermore, we applied the bias of −4.5 V on the gate electrode with other electrodes grounded in order to investigate the contribution of only bias on the electrical properties. The bias is applied to MOSFETs without irradiation and measurement procedure is the same as the case of irradiation experiment. Figure 6 shows the *I*_D_–*V*_G_ curves of the MOSFETs before applying bias and after 24 h aging. This aging time corresponds to the time for a cumulative dose of 240 kGy in our irradiation condition. The *I*_D_–*V*_G_ curve of the aged sample is almost consistent with that of the pristine one, except for a slightly increased leakage current. Hence, only the application of the gate bias does not significantly affect the electrical properties. Accumulation and annealing of trapped charges observed in this study are attributed to a synergy effect between the gate bias and irradiation.

## 5. Conclusions

Electrical properties of prototype SiC MOSFETs irradiated at various gate biases were characterized. Threshold voltages (*V*_th_) for un- and negative-biased up to −4.5 V samples slightly shift toward the negative voltage side, due to irradiation. A further increasing bias was observed negatively up to −9 V, *V*_th_ shift more negatively. Moreover, a further negative shift of *V*_th_ was observed for the positive bias of 2.25 V. Positive charge densities trapped in the gate oxide of un- and positive-biased samples increased with increasing dose, while no significant increase was observed for negative- biased samples of −2.25 and −4.5 V. The characteristic parameter for accumulation of holes in the gate oxide, σ_p_*J*_p_, was lower for these negative biased samples compared with the unbiased case. Further increasing the negative bias up to −9 V, σ_p_*J*_p_ is not significantly varied. This indicates that negative bias and *E*_ox_ suppress the movement of holes toward traps located close to the SiC/SiO_2_ interface and only a portion of holes generated by irradiation were trapped. In contrast, there is not as large a difference in the parameter for annihilation of holes trapped in the oxide, σ_n_*J*_n_, among −2.25 and −4.5 V biased and unbiased samples. For −9 V biased case, no annihilation effect was recognized. These results indicate that trapped holes were predominantly annealed by electrons coming from the SiC side. Application of negative gate biases to SiC MOSFETs during irradiation by which |*E*_ox_| within approximately 0.5 MV/cm was generated, suppresses accumulation of holes in the gate oxide.

## References

[1] Nagatani K., Kiribayashi S., Okada Y., Otake K., Yoshida K., Tadokoro S., Nishimura T., Yoshida T., Koyanagi E., Fukushima M. (2013). Emergency response to the nuclear accident at the Fukushima Daiichi Nuclear Power Plants using mobile rescue robots. J. Field. Robot..

[2] Cho J., Choi Y., Jeong K. (2014). Monitoring performance of the cameras under the high dose-rate gamma ray environments. Health Phys..

[3] Cho H., Woo T. (2015). Project strategy for clean-up of sedimentry radioactive material in Fukushima bay areas using snake-like robotics. Nucl. Tec. Rad. Prot..

[4] Dodd P.E., Shaneyfelt M.R., Schwank J.R., Felix J.A. (2010). Current and future challenges in radiation effects on CMOS electronics. IEEE Trans. Nucl. Sci..

[5] Fleetwood D.M. (2013). Total ionizing dose effects in MOS and low-dose-rate-sensitive linear-bipolar devices. IEEE Trans. Nucl. Sci..

[6] Mitomo S., Matsuda T., Murata K., Yokoseki T., Makino T., Takeyama A., Onoda S., Ohshima T., Okubo S., Tanaka Y. (2017). Optimum structures for gamma-ray radiation resistant SiC-MOSFETs. Phys. Status Solidi A.

[7] Matsuda T., Yokoseki T., Mitomo S., Murata K., Makino T., Abe H., Takeyama A., Onoda S., Tanaka Y., Kandori M. (2016). Change in characteristics of SiC MOSFETs by gamma-ray irradiation at high temperature. Mater. Sci. Forum..

[8] Ohshima T., Yokoseki T., Murata K., Matsuda T., Mitomo S., Abe H., Makino T., Onoda S., Hijikata Y., Tanaka Y. (2016). Radiation response of silicon carbide metal–oxide–semiconductor transistors in high dose region. Jpn. J. Appl. Phys..

[9] Akturk A., McGarrity J.M., Potbhare S., Goldsman N. (2012). Radiation effects in commercial 1200 V 24 A silicon carbide power MOSFETs. IEEE Trans. Nucl. Sci..

[10] Takeyama A., Matsuda T., Yokoseki T., Mitomo S., Murata K., Makino T., Onoda S., Okubo S., Tanaka Y., Kandori M. (2016). Improvement of radiation response of SiC MOSFETs under high temperature and humidity conditions. Jpn. J. Appl. Phys..

[11] Murata K., Mitomo S., Matsuda T., Yokoseki T., Makino T., Onoda S., Takeyama A., Ohshima T., Okubo S., Tanaka Y. (2017). Impacts of gate bias and its variation on gamma-ray irradiation resistance of SiC MOSFETs. Phys. Status Solidi A.

[12] Zhang T., Allard B., Bi J. (2018). The synergetic effects of high temperature gate bias and total ionization dose on 1.2 kV SiC devices. Microelectron. Reliab..

[13] Messenger G.C., Steele E.J., Neustadt M. (1965). Displacement damage in MOS transistors. IEEE Trans. Nucl. Sci..

[14] Anant G.S. (1983). Characterization of annealing of Co60 gamma-ray damage at the Si/SiO_2_ interface. IEEE Trans. Nucl. Sci..

[15] Tallon R.W., Ackermann M.R., Kemp W.T., Owen M.H., Saunders D.P. (1985). A Comparison of ionizing radiation damage in MOSFETs from cobalt-60 gamma rays, 0.5 to 22 MeV protons and 1 to 7 MeV electrons. IEEE Trans. Nucl. Sci..

[16] Schwank J.R., Shaneyfelt M.R., Fleetwood D.M., Felix J.A., Dodd P.E., Paillet P., Ferlet-Cavrois V. (2008). Radiation effects in MOS oxides. IEEE Trans. Nucl. Sci..

[17] Oldham T.R., McLean F.B. (2003). Total ionizing dose effects in MOS oxides and devices. IEEE Trans. Nucl. Sci..

[18] Zhang C., Zhang E., Fleetwood D.M., Schrimpf R.D., Dhar S., Ryu S., Shen X., Pantelides S.T. (2011). Effects of bias on the irradiation and annealing responses of 4H–SiC MOS devices. IEEE Trans. Nucl. Sci..

[19] Faigon A., Lipovetzky J., Redin E., Krusczenski G. (2008). Extension of the measurement range of MOS dosimeters using radiation induced charge neutralization. IEEE Trans. Nucl. Sci..

[20] Sambuco Salomone L., Faigón A., Redin E. (2015). Numerical Modeling of MOS dosimeters under switched bias irradiations. IEEE Trans. Nucl. Sci..

[21] Fleetwood D.M., Winokur P., Riewe L. (1990). Predicting switched-bias response from steady-state irradiations MOS transistors. IEEE Trans. Nucl. Sci..

[22] Faigon A., Garcia Inza M., Lipovetzky J., Redin E., Carbonetto S., Sambuco Salomone L., Berbeglia F. (2014). Experimental evidence and modeling of non-monotonic responses in MOS dosimeters. Radiat. Phys. Chem..

[23] Fleetwood D.M., Reber R.A., Winokur P.S. (1992). Trapped-hole annealing and electron trapping in metal-oxide-semiconductor devices. Appl. Phys. Lett..

[24] Haller G., Knoll M., Bräunig D., Wulf F., Fahrner W.R. (1984). Bias-temperature stress on metal-oxide-semiconductor structures as compared to ionizing irradiation and tunnel injection. J. Appl. Phys..

[25] McWhorter P.J., Winokur P.S. (1986). Simple technique for separating the effects of interface traps and trapped-oxide charge in metal-oxide-semiconductor transistors. Appl. Phys. Lett..

[26] Sze S.M., Ng K.K. (2007). Physics of Semiconductor Devices.

[27] Yoshioka H., Senzaki J., Shimozato A., Tanaka Y., Okumura H. (2014). Effects of interface state density on 4H–SiC n-channel field-effect mobility. Appl. Phys. Lett..

[28] Boesch H.E., McLean F.B., Benedetto J.M., McGarrity J.M. (1986). Saturation of threshold voltage shift in MOSFET's at high total dose. IEEE Trans. Nucl. Sci..

[29] Oldham T.R., Lelis A.J., McLean F.B. (1988). Spatial Dependence of trapped holes determined from tunneling analysis and measured annealing. IEEE Trans. Nucl. Sci..

[30] Rozen J., Dhar S., Dixit S.K., Afanas’ev V.V., Roberts F.O., Dang H.L., Wang S., Pantelides S.T., Williams J.R., Feldman L.C. (2008). Increase in oxide hole trap density associated with nitrogen incorporation at the SiO_2_/SiC interface. J. Appl. Phys..

[31] Lelis A.J., Boesch H.E., Oldham T.R., McLean F.B. (1988). Reversibility of trapped hole annealing. IEEE Trans. Nucl. Sci..

[32] Suzuki S., Harada S., Kosugi R., Senzaki J., Cho W., Fukuda K. (2002). Correlation between channel mobility and shallow interface traps in SiC metal–oxide–semiconductor field-effect transistors. J. Appl. Phys..

[33] Potbhare S., Goldsman N., Pennington G., Lelis A., McGarrity J.M. (2006). Numerical and experimental characterization of 4H–silicon carbide lateral metal–oxide–semiconductor field-effect transistor. J. Appl. Phys..

[34] Potbhare S., Goldsman N., Lelis A., McGarrity J.M., McLean F.B. (2008). A physical model of high temperature 4H–SiC MOSFETs. IEEE Trans. Electron. Devices.

[35] Sometani M., Okamoto D., Harada S., Ishimori H., Takasu S., Hatakeyama T., Takei M., Yonezawa Y., Fukuda K., Okumura H. (2016). Threshold-voltage instability in 4H–SiC MOSFETs with nitrided gate oxide revealed by non-relaxation method. Jpn. J. Appl. Phys..

[36] JEDEC (2015). Procedure for Wafer-Level DC Characterization of Bias Temperature Instabilities.

